# Chironomids (Diptera) from Central European stream networks: new findings and taxonomic issues

**DOI:** 10.3897/BDJ.12.e136241

**Published:** 2024-12-27

**Authors:** Bernadett Boóz, Zsolt Kovács, Bea Bartalovics, Pál Boda, Marko Miliša, Bálint Pernecker, Petr Pařil, Tomasz Rewicz, Anna Boglárka Simon, Zoltán Csabai, Arnold Móra

**Affiliations:** 1 University of Pécs, Faculty of Sciences, Department of Hydrobiology, Ifjúság útja 6, Pécs, Hungary University of Pécs, Faculty of Sciences, Department of Hydrobiology, Ifjúság útja 6 Pécs Hungary; 2 HUN-REN Centre for Ecological Research, Institute of Aquatic Ecology, Bem square 18/C, Debrecen, Hungary HUN-REN Centre for Ecological Research, Institute of Aquatic Ecology, Bem square 18/C Debrecen Hungary; 3 University of Zagreb, Faculty of Science, Department of Biology, Ravnice 48, Zagreb, Croatia University of Zagreb, Faculty of Science, Department of Biology, Ravnice 48 Zagreb Croatia; 4 Masaryk University, Faculty of Science, Department of Botany and Zoology, Kotlářská 2, Brno, Czech Republic Masaryk University, Faculty of Science, Department of Botany and Zoology, Kotlářská 2 Brno Czech Republic; 5 University of Lodz, Faculty of Biology and Environmental Protection, Department of Invertebrate Zoology and Hydrobiology, Stefana Banacha 12/16, Łódź, Poland University of Lodz, Faculty of Biology and Environmental Protection, Department of Invertebrate Zoology and Hydrobiology, Stefana Banacha 12/16 Łódź Poland; 6 HUN-REN Balaton Limnological Research Institute, Klebelsberg Kuno 3, Tihany, Hungary HUN-REN Balaton Limnological Research Institute, Klebelsberg Kuno 3 Tihany Hungary; 7 Eötvös Loránd University, Department of Environmental and Landscape Geography, Pázmány Péter sétány 1/C, Budapest, Hungary Eötvös Loránd University, Department of Environmental and Landscape Geography, Pázmány Péter sétány 1/C Budapest Hungary

**Keywords:** non-biting midges, GBIF, integrative taxonomy, checklist, new records, Hungary, Croatia, Czechia, stream

## Abstract

**Background:**

Chironomidae, with over 7,300 described species, are amongst the most diverse and abundant insect families in freshwater ecosystems worldwide. Chironomids are known for their widespread distribution from various water types. The level of documentation of chironomid fauna varies considerably amongst European countries, with more comprehensive knowledge for Western Europe compared to other regions. Despite the recent extensive sampling effort and the increasing number of available data, the chironomid fauna of Central European countries still remains poorly known.

**New information:**

This study contributes to the knowledge of chironomid fauna in three river catchments in Croatia, Hungary and Czechia. A combination of morphological and molecular techniques was employed, with a focus on larvae, although pupae and exuviae were also examined. We found 207 taxa, amongst which 170 were identified to species level. In Croatia, 14 species were recorded for the first time and two species were newly recorded in Czechia. DNA barcoding of 31 specimens resulted in 23 BINs, including eight new ones to BOLD. We provided detailed notes on taxa with taxonomic problems and/or morphological peculiarities. Our results highlight that extensive studies conducted in relatively small areas and a limited range of habitats (only streams in hilly regions) can remarkably contribute to the local and global knowledge on Chironomidae fauna, especially when the taxonomically difficult and often problematic larvae are investigated.

## Introduction

The Diptera family Chironomidae (also known as non-biting midges) comprise the most diverse and frequently the most abundant insect group found in freshwaters. Although larvae of some species can be semi-aquatic or terrestrial, the majority are strictly aquatic (Armitage et al. 1995). The immature stages of chironomids can inhabit a wide range of water types, from thin water film on glaciers to plant-held waters, streams, rivers, lakes, ponds, reservoirs, brackish waters and shoreline habitats (Armitage et al. 1995). Besides their widespread distribution, the species richness of Chironomidae is usually amongst the highest of aquatic insect families, occasionally exceeding 100 species per site in many aquatic habitats (Ferrington 2008).

Globally, approximately 7,300 chironomid species are currently known (Pape et al. 2011), but the estimated number of species may be around 15,000 (Armitage et al. 1995). According to Fauna Europaea (de Jong et al. 2014, currently not accessible), nearly 1,300 species have been recorded only from Europe. The available information on chironomid fauna varies considerably amongst European countries, with more comprehensive knowledge for Western Europe compared to other regions (Bitušík et al. 2020). In Central European countries, the chironomid fauna is still limitedly known, despite recent extensive collections and an increasing amount of available data.

The present study provides a contribution to the knowledge on chironomid fauna of three Central European countries by presenting results from detailed investigations conducted within three selected river catchments representing Croatia, Hungary and Czechia. In this research, we primarily focused on larvae, but pupae and exuviae were also included occasionally and molecular techniques have been applied for certain taxa to achieve precise identification. We provide here the first records of some species in Croatia and Czechia. Notes are given for selected taxa of taxonomic interest.

## Materials and methods

### Study area

**Figure 1.**
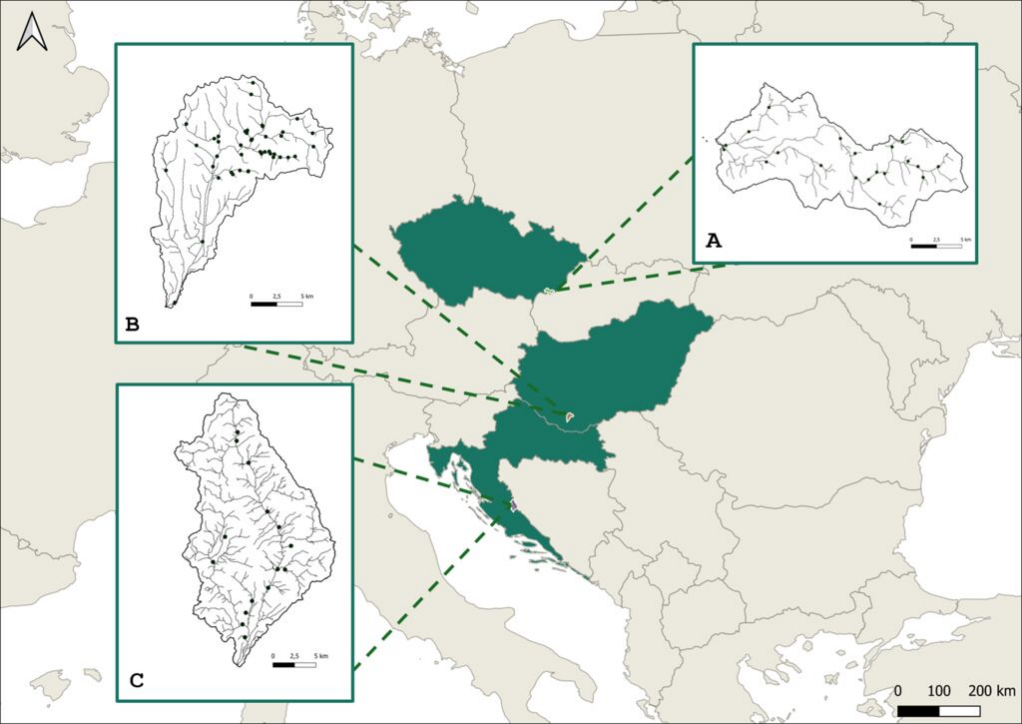
Map of the study area. **A** Velička River catchment, Czechia; **B** Bükkösdi-víz River catchment, Hungary; **C** Butižnica River catchment, Croatia.

Our investigations cover three river catchment areas from three Central European countries (Fig. 1) representing three European ecoregions. Bükkösdi-víz (BUK) is located in the south-western part of Hungary (Pannonian ecoregion with Continental climate) with an area of 185 km^2^ and forms a sub-catchment of Fekete-víz within the drainage area of the Drava River. The area is characterised by the hilly surroundings of Mecsek Mountains (sampling sites were located between 104–245 m a.m.s.l.). The Butižnica River (BUT) catchment is situated in the Dinaric Mountains (sampling sites were located between 235–591 m a.m.s.l.), North-Central Dalmatia, Croatia (Balkanic ecoregion, Mediterranean climate). Its drainage area is around 225 km^2^ and belongs to the Krka River catchment. The Velička River (VEL) is a sub-catchment of the Morava River and it is located in the South Moravian part of Czechia in the White Carpathian Mountains (Continental ecoregion, humid continental climate). Velička catchment area of 172 km^2^ was included in the sampling design of this project (sampling sites were located between 174–446 m a.m.s.l.) (Fig. 1).

### Sampling, sample processing and identification

Chironomidae larvae were collected using multihabitat sampling methods during two campaigns (Suppl. material 1): one in 2018–2019 at the Bükkösdi-víz catchment and another in 2021 at all catchments. Additionally, at some sites in Bükkösdi-víz catchment, floating chironomid pupal exuviae were collected from the water surface using a hand net (frame 25 × 25 cm, mesh size 250 µm) and a plastic tray in 2021.

In case of larvae and pupae, all individuals were identified from samples containing less than 300 individuals, while subsamples of ca. 300 individuals were taken from larger samples. We used multi-level identification methods that included: separating specimens based on macroscopic characteristics; conducting detailed investigations without preparation and using light microscope; and mounting specimens on microscope slides for examination at higher magnification. Exuviae were mounted on microscope slides in every instance. For morphological identification of larvae and pupae/exuviae, multiple keys were used. For further details, see Suppl. material 1.

In addition to morphological identification, a subset of larvae underwent molecular analysis using a traditional DNA barcoding protocol (Suppl. material 1). All sequences were uploaded to the Barcode of Life Data Systems (BOLD; Ratnasingham and Hebert 2007).

## Data resources

Over 140,000 chironomid individuals were collected from 456 samples. A total of 207 taxa identified to at least genus level were documented, belonging to five subfamilies (32 Tanypodinae, 8 Diamesinae, 3 Prodiamesinae, 103 Orthocladiinae, 61 Chironominae) and 74 genera, providing 5,481 occurrence records. A total of 170 taxa were identified to species level (82% of all taxa), accounting for 3,613 occurrence data (66% of all records). We recorded 117 taxa (93 species) from Croatia, 127 taxa (102 species) from Hungary and 128 taxa (104 species) from Czechia. Across the three countries, *Micropsectra* sp. (representing different species) provided the highest number of occurrence records amongst taxa, while *Parametriocnemusstylatus* (Spärck, 1923) accounted for the highest number of species-level occurrences, ranking first in both Croatia and Hungary. However, in Czechia, *Polypedilumconvictum* (Walker, 1856) was the most frequent species.

All our records have been uploaded to the Global Biodiversity Information Facility (GBIF) from 78 localities with georeferenced coordinates, available at https://www.gbif.org/dataset/45b4c600-6d61-4eeb-b9a6-6d07e0cdcd1a (Boóz et al. 2024a).

### Newly-recorded Chironomidae species

The first occurrence of 14 species, *Macropelopianotata* (Meigen, 1818), *Eukiefferiellabrevicalcar* (Kieffer, 1911), *Eukiefferiellaclaripennis* (Lundbeck, 1898), *Hydrobaenusdistylus* (Kieffer, 1915), *Krenosmittiacamptophleps* (Edwards, 1929), *Orthocladiuscalvus* Pinder, 1985, *Orthocladiusluteipes* Goetghebuer, 1938, *Orthocladiussaxosus* (Tokunaga, 1939), *Parakiefferiellatriquetra* (Pankratora, 1970), *Rheocricotopusunidentatus* Saether & Schnell, 1988, *Rheosmittiaspinicornis* (Brundin, 1956), *Microtendipesrydalensis* (Edwards, 1929), *Paratendipesnudisquama* (Edwards, 1929) and *Endochironomusdonatoris* Shilova, 1974 were recorded in Croatia. Additionally, the first occurrence of two species, *Rheocricotopusunidentatus* Saether & Schnell, 1988 and *Microtendipesbritteni* (Edwards, 1929) were registered in Czechia.

### Molecular identifications

DNA was extracted from a total of 31 specimens (14 from Hungary, 10 from Croatia and 7 from Czechia) that were uploaded to BOLD. Sequences had a median length of 658 bp, ranging from 569 to 659 bp, meaning all the sequences were high-quality barcodes (≥ 500 bp) being assigned to a Barcode Index Number (BIN; Ratnasingham and Hebert 2013). The 31 COI-sequences were assigned to 23 BINs, including eight unique BINs that are new to BOLD. Half of the specimens were identified to species level; however, in the case of 16 specimens, there were no matches passing the 1.6% threshold, preventing conclusive species-level identification.

### Notes on selected taxa

In this section, we summarise detailed information on taxa with taxonomic issues and/or morphological peculiarities. Our aim was not to provide full taxonomic descriptions; therefore, we only highlight the relevant differential characteristics.

*Clinotanypus* – In most parts of Europe, *C.nervosus* can be found (Andersen et al. 2013), but from Russia and Romania, larvae identified as *C.pinguis* have also been recorded (Botnariuc and Cure 1999). The difference between the two species is that *C.nervosus* has a 5-tooth ligula, while *C.pinguis* has ligula with 6–7 teeth (Janecek 1998, Botnariuc and Cure 1999, Klink and Moller Pillot 2003, Andersen et al. 2013). However, *C.nervosus* is regarded as the only western Palaearctic representative of the genus, while *C.pinguis* is widely distributed in North America (Andersen et al. 2013) and, to our best knowledge, has never been found in the Palaearctic as adult. In our study, *Clinotanypus* larvae were found in Hungary and all of them had 6-toothed ligula. DNA barcoding revealed that this morphotype belongs to *C.nervosus*, proving that two morphotypes of this species exist and the new morphotype can be dominant in some populations. Furthermore, our results raise the possibility that the occurrence of *C.pinguis* in Europe might be based on misidentification.

*Conchapelopia* – In our study, larvae were identified to species level only when associated pupae were available. We distinguished two pupal morphotypes in our material: pupae of *C.melanops* are well recognisable, while there are small differences between *C.pallidula* and *C.triannulata*, species representing the other morphotype. In Hungary, the morphology of the collected specimens clearly fits the description of *C.triannulata* (Michiels and Spies 2002), which was also found in a previous study close to the Bükkösdi-víz catchment (Boóz and Móra 2020). However, the Croatian specimens were not unambiguously separable and were identified as a species pair.

*Thienemannimyia* – The species of the genus are not or hardly distinguishable as either larvae or pupae (Langton and Visser 2003, Vallenduuk and Moller Pillot 2007). In our study, larvae were morphologically identified to genus level, while one pupa from Czechia was identified as *T.carnea*, and one pupa from Croatia as *T.lentiginosa*/*laeta*. DNA barcoding was performed for the latter pupa and two larvae, but no match was found in BOLD systems in either case.

*Zavrelimyia* – According to the broadened generic conception of *Zavrelimyia*, the genus contains more former genera as subgenera (Silva and Ekrem 2016). In our study subgenera *Zavrelimyia* s. str. and Zavrelimyia (Paramerina) occurred, with larvae identifiable only to subgenera by morphological characters. Some pupae/exuviae were identified as Z. (Z.) barbatipes and as Z. (P.) cingulata. The pupal form *Paramerina* spec-Griechenland (Langton and Visser 2003) was found in Croatia and sequences from DNA barcoding matched (100% and 98.48% similarity) with one sequence under the name *Zavrelimyiadivisa* in BOLD Systems. However, these sequences differed more than 9% from other sequences of *Z.divisa*, which questions the correctness of species identification. The clear morphological differences between Z. (P.) divisa and *P.* spec-Griechenland and the inconsistencies in molecular results suggest that the pupal form represents a different species.

*Diamesa* – There are many difficulties in separating *Diamesa* species as larvae (e.g. Janecek (1998), Rossaro and Lencioni (2015)) and as pupae (Langton and Visser 2003) by morphological characters. In our study, identification was possible only to the species group level in many cases (*D.cinerella*-Gr., *D.zernyi*-Gr.). Amongst *D.insignipes/cinerella/tonsa*, only pupae with very typical characters (see Langton and Visser (2003)) were identified as *D.insignipes* or as *D.tonsa*. In the case of larvae, *D.tonsa* was relatively easily recognisable, based on the colouration of the head (Rossaro and Lencioni 2015), but *D.insignipes* was only accepted when DNA barcoding confirmed the species identity (one specimen from Croatia and two specimens from Czechia).

*Monodiamesa* – Some larvae were found in Bükkösdi-víz catchment, Hungary. Only one species, *M.nitida* has been recorded in the country (Móra and Dévai 2004), but neither the larva nor the pupa of this species is known (Schmid 1993, Langton and Visser 2003). The other species that potentially might occur in Hungary is *M.bathyphila*, a widespread species in Europe. However, DNA barcoding revealed a high distance between *M.bathyphila* and our studied individual (only 89.74% similarity), suggesting that the collected specimens might belong to *M.nitida*.

*Chaetocladius* – The morphological identification of the larvae of this genus is only possible at species group level (Janecek 1998, Orendt and Bendt 2021), whereas species can be distinguished as pupal exuviae (Langton and Visser 2003). For this reason, in our study, only exuviae were identified to species (*C.piger*, *C.laminatus*). One pupa was identified as a pupal form (*Chaetocladius* pe1) because not all characters were clearly recognisable, but, most probably, it was an atypical individual of *C.piger*.

*Corynoneura* – From one site in Hungary, we identified the larval morphotype as C.cf.antennalis (Schmid 1993, Klink and Moller Pillot 2003), although this name is regarded as a synonym of *C.celeripes* by some authors (Evenhuis and Pape 2024), but questioned by other experts (Moller Pillot 2013). Accordingly, we used the name *C.antennalis* (as cf.) in our dataset. A larval morphotype (*Corynoneura* sp. BUT) was collected from many sites in Croatia that closely resembles *C.lobata* (three median teeth, strongly reduced first lateral teeth), but the central median tooth on the mentum is as large as the other median teeth (see *Corynoneura* species A in Cranston (1982)).

*Cricotopus* – Despite the available keys for *Cricotopus* species (e.g. Hirvenoja (1973), Langton and Visser (2003), Cuppen and Tempelman (2018)), specimens of this genus were identified to species level only occasionally due to the little morphological differences between species in many cases.

*Epoicocladius* – The Palaearctic *E.ephemerae* and Nearctic *E.flavens* are currently recognised as separate species (e.g. Ashe and O’Connor (2012), Andersen et al. (2013)); however, some authors suggest they are synonyms (e.g. Evenhuis and Pape (2024)). Accordingly, we used the name *E.ephemerae* for our records. Larvae collected in Butižnica River catchment were somewhat different from those in other populations, as the setae on body segments were remarkably less numerous and paler. DNA barcoding resulted in no match with any *Epoicocladius* sequences, raising the possibility that these larvae represent a different species within the genus.

**Figure 2.**
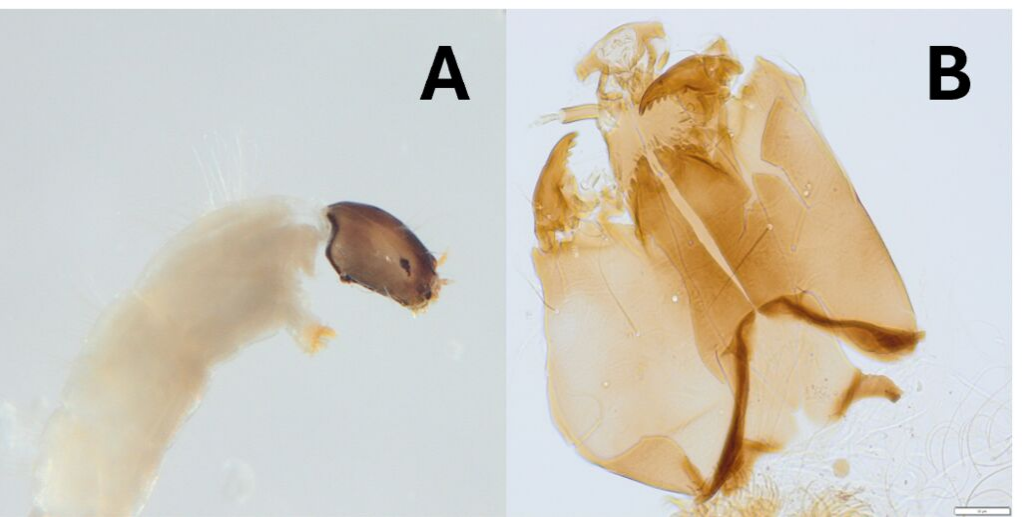
Larvae of *Eukiefferiella* sp. BUT. **A** thorax with dense hair-like setae and colour of the head; **B** details of the head from ventral view.

*Eukiefferiella* – In the case of *E.devonica*, larvae were identified to species only as pupae and to species group as larvae. *Eukiefferiellaminor* and *E.fittkaui* were separated neither as larvae nor as pupae due to very little morphological differences (Langton and Visser 2003, Orendt and Bendt 2021). A strange larval morphotype (*Eukiefferiella* sp. BUT) was found in the Butižnica River catchment: its morphology fits the generic description of *Eukiefferiella* (Andersen et al. 2013), but the thorax is covered with dense, relatively long (approx. half the width of the thoracic segment) setae (Fig. 2), which is unique in the genus. In the Velička River catchment, a peculiar pupa (*Eukiefferiella* sp. VEL) was collected. In the key of Langton and Visser (2003), the specimen could be identified as either *E.claripennis* or *E.brevicalcar* due to the swollen base of the thoracic horn, but the length of its apical filament separates this morphotype from both species (longer than in *E.claripennis*, but shorter than in *E.brevicalcar*). Although the pattern of the hooks on abdominal segments resembles *E.claripennis*, it is not clear whether it is a new pupal form or a non-typical representative of one or the other species.

*Krenosmittia* – The two common species of the genus (*K.boreoalpina* and *K.camptophlebs*) can be separated by the length of the 1^st^ antennal segment and the head (Schmid 1993), but this separation is considered as tentative (Janecek 1998). In the Croatian material, the dimensions of the head and the antenna fell in the range of *K.camptophleps* and pupae found here also confirmed the species identity. However, in the Czech material, only larvae occurred and their dimensions were ambiguous; therefore, we identified these specimens only to genus level.

*Limnophyes* – Larvae of the genus are morphologically indistinguishable (e.g. Janecek (1998), Orendt and Bendt (2021)). DNA barcoding performed on one of the many morphologically identical larvae from the Bükkösdi-víz catchment revealed that likely they belong to *L.minimus*.

*Metriocnemus* – Larvae of the genus are hardly distinguishable due to poorly-known morphological differences (Orendt and Bendt 2021) and many species with undescribed larvae (Moller Pillot 2013). DNA barcoding was performed on a larva from the Bükkösdi-víz catchment, but there was no match with valid species in BOLD systems, although the generic status was confirmed.

Orthocladius – Larvae and pupae belonging to the subgenus Orthocladius s. str. were identified to species level only occasionally due to unclear taxonomy and little morphological differences between the species (Cuppen and Tempelman 2022). Nearly all larvae and pupae belonging to the subgenus Euorthocladius were separated to species using multiple keys (Schmid 1993, Janecek 1998, Klink and Moller Pillot 2003, Cuppen and Tempelman 2022). One larva was morphologically different from the identifiable ones and either the DNA barcoding resulted in no match in BOLD Systems. Accordingly, this larva represents a new morphotype and a species that, at least as larvae, has not yet been described. In the subgenus Symposiocladius, larvae were identified as O. (S.) lignicola and as O. (S.) holsatus based on the L4 hair tuft and morphology of the mentum (Cuppen and Tempelman 2022). The occurrence of O. (S.) lignicola in Bükkösdi-víz catchment was also confirmed by pupae (Langton and Visser 2003). However, both larval types might represent other species as well, for example, the larva of O. (S.) ruffoi is not known (Cuppen and Tempelman 2022). Larvae from the Butižnica River catchment closely resembled O. (S.) holsatus, but the L4 hair tuft was much shorter than it is depicted in Cuppen and Tempelman (2022); accordingly, they only were identified to subgenus level.

*Parametriocnemus* – Larvae from all catchments were identified as *P.stylatus*, based on their morphology (e.g. Janecek (1998)) and co-occurrence of conspecific pupae (Langton and Visser 2003). However, DNA barcoding of a larva from the Bükkösdi-víz catchment resulted in no match with *P.stylatus*, suggesting that it is rather a species complex (see Orendt and Bendt (2021)). Some pupae collected in the Velička River catchment proved to be *Parametriocnemus* Pe1; DNA barcoding also resulted in no match in BOLD systems in this case.

*Paraphaenocladius* – From the Velička River catchment, larvae were identified as *P.impensus* (Klink and Moller Pillot 2003), but only pupae of *P.irritus* (Langton and Visser 2003) were found at the same site. The larvae of *P.irritus* is not known/not included in the keys available for European *Paraphaenocladius* species (e.g. Klink and Moller Pillot (2003), Orendt and Bendt (2021)). Accordingly, the possibility arises that the larvae of *P.impensus* and *P.irritus* are morphologically identical or hardly separable and only the latter species was collected in Czechia.

**Figure 3.**
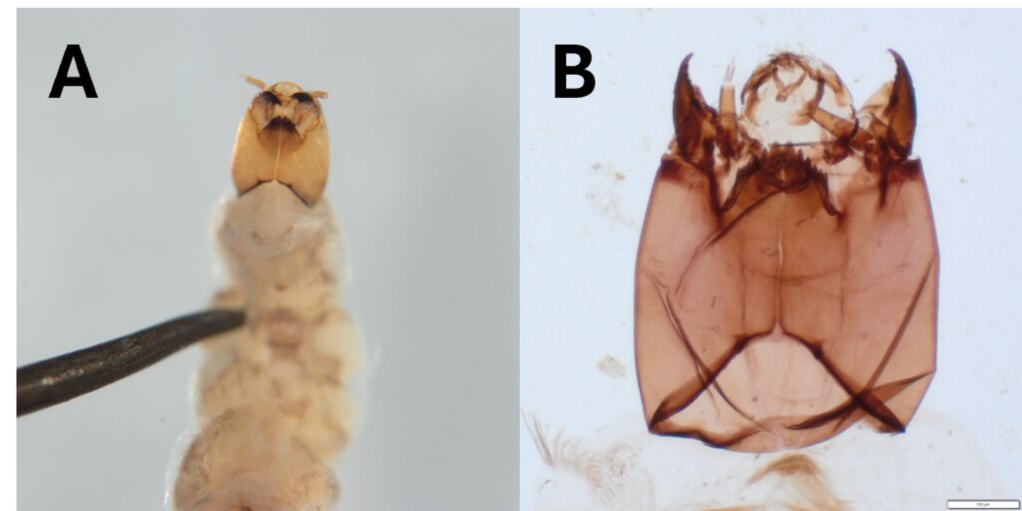
Larvae of *Rheocricotopus* sp. VEL. **A** head and thorax from ventral view; **B** details of the head.

*Rheocricotopus* – At multiple sites in the Velička River catchment, a morphotype (*Rheocricotopus* sp. VEL) was found (Fig. 3), which has the general characteristics of the genus, but does not resemble any species with currently known larva or pupa (Schmid 1993, Janecek 1998, Langton and Visser 2003, Orendt and Bendt 2021).

*Tvetenia* – In the Butižnica River catchment, two larval morphotypes were found that differed from any species in the genus (Schmid 1993, Janecek 1998, Langton and Visser 2003, Orendt and Bendt 2021). *Tvetenia* sp. A is a known larval form (e.g. Cranston (1982), Schmid (1993), Klink and Moller Pillot (2003)) and was suggested to be linked with various species (Janecek 1998, Klink and Moller Pillot 2003, Orendt and Bendt 2021). *Tvetenia* sp. BUT clearly belongs to this genus, based on the morphology of the head and the presence of long setae on body segments, but does not resemble any other species with known larva.

**Figure 4.**
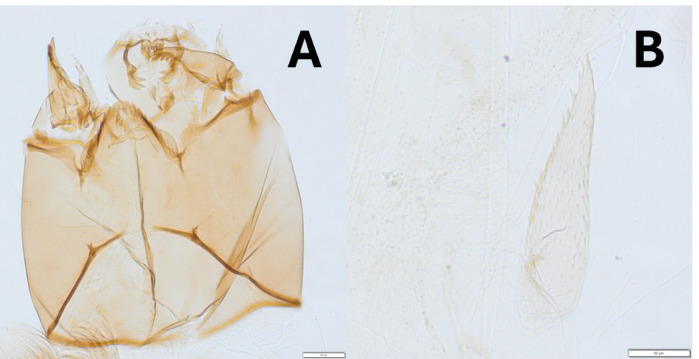
Larvae of Orthocladiinae Gen. sp. III. **A** details of the head; **B** thoracic horn visible in pharate larvae.

Genus unknown – The morphology of some larvae from the Velička River catchment clearly matches with the taxon Orthocladiinae sp. “Berka vor dem Hainich, Thüringen” in Orendt and Bendt (2021). Our results proved that the distribution of this taxon is not limited to Germany. Orthocladiinae larvae and a pharate larvae with unique morphological characteristics were found in Bükkösdi-víz catchment (Orthocladiinae Gen. sp. III.). Neither larval nor pupal characteristics (Fig. 4) resemble any species or genera included in the keys used in our work. Some larval characters suggest that this morphotype belongs to *Parakiefferiella*, but the thoracic horn is completely different from that characteristic for the genus. DNA barcoding also resulted in no match in BOLD Systems.

*Chironomus* – Based on DNA barcoding, a larva from the Bükkösdi-víz catchment identified earlier as *C.entis* by morphological characters, proved to be *C.plumosus*. As sequences can also be found for *C.entis* in the BOLD Systems, we accept that this result is correct. In our larva, the upper surface of the antennal pedestal was completely light brown, which is a distinctive character for *C.entis* according to Vallenduuk (2017). Moreover, the dimensions of the parts of the head fell in the range of this species, but it must be noted that these characters can vary and overlap between the species. According to Orendt and Spies (2012), *C.plumosus* can be recognised by a distinct dark streak between the antennal pedestal and the upper eyespot. Our specimen proved that this pigmentation could spread further on the upper surface of the antennal pedestal. Accordingly, all characters used to separate the two species may overlap and should be regarded with caution. DNA barcoding performed on a larva identified as *C.bernensis* resulted in the closest match with a sequence under the name *C.annularius*. However, in this BIN, there are also specimens under the name *C.bernensis* MOTU7, which was analysed by Pfenninger et al. (2007), with the result that the nominal species *C.bernensis* might be a cryptic species complex. However, the taxonomy of *C.annularius* is also not clear with more biological species mentioned under this name by various authors (Spies and Sæther 2004). Nevertheless, the two larval morphotypes that represent these names can clearly be separated by the presence of lateral gills on abdominal segment VII in *C.annularius*, while these gills lack in *C.bernensis* (e.g. Orendt and Spies (2012), Vallenduuk (2017)). As the larvae we found had no lateral gills on segment VII, accordingly we listed them as *C.bernensis*.

*Phaenopsectra* – Larvae from the Bükkösdi-víz and Velička River catchments were identified as *P.flavipes* (Vallenduuk 2017), which was confirmed by DNA barcoding of an associated pupa. Another larval form was collected in the Butižnica River catchment, with the mentum and mandible being identical with those depicted as *Phaenopsectra* sp. in Andersen et al. (2013).

*Polypedilum* – Larvae identified as *P.convictum* from the Butižnica River catchment had elongate anal tubules which is unusual for this species (triangular tubules are characteristic, Vallenduuk (2017)). It is not clear if this difference is a population level variability or indicates a different species.

*Micropsectra* – We had difficulties with the identification of a larval Tanytarsini morphotype (cf. *Micropsectra* sp.) from Bükkösdi-víz catchment. The bifid premandible suggested *Micropsectra*, but the remarkably small body size, the lack of spur on antennal pedestal and the small number of claws on posterior parapods rather resemble *Tanytarsus*. The separation of the two genera is very difficult and probably the best character is the shape of the premandible (Andersen et al. 2013). Based on the bifid premandible, our larvae most probably belong to *Micropsectra*. In addition, co-occurring small *Micropsectra* pupae were also collected. These pupae resemble those of *M.notescens*, but are much smaller and less pigmented. The similar size of the larvae and pupae raises the possibility that they might represent the same species. The pupal morphotype *Micropsectra* sp. BUK1 was collected at one site in the Bükkösdi-víz catchment. This morphotype is most similar to *M.auvergnensis* due to the shagreen on the pleura of segments II-V, but, in the case of the latter species, pleura of segments II-VII have shagreen. Furthermore, male hypopygium was observable in one pupa and it clearly differed from that of *M.auvergnensis* (Reiss 1969). Further studies are in progress to clarify the taxonomic status of this species.

## Checklists

### Chironomidae from Central Europe

#### Chironomidae

DAB5B22E-3830-523F-B4C6-F8358BA54902

https://www.gbif.org/dataset/45b4c600-6d61-4eeb-b9a6-6d07e0cdcd1a

## Discussion

The first comprehensive species list of Chironomidae of Croatia was published by Čerba et al. (2020), presenting 239 species. Since then, more papers have been published (Suppl. material 2) bringing the number of species to 245. Our study added 14 new species to the Croatian fauna raising the number of species to 259. The fact that the new species came from a single, relatively small area, indicates that the chironomid species list of the country is far from complete.

The latest updated checklist of Chironomidae of Czechia was published by Bitušík and Brabec (2009) and it included 271 species. Since then, multiple papers (Suppl. material 2) contributed with an additional 104 species to the fauna. Our study contributed to the species list with two species raising the number of species to 377.

The first comprehensive checklist of Hungarian chironomid fauna was published by Móra and Dévai (2004) listing 228 species. Since then, more than 20 faunistic studies were published containing new species to the Hungarian fauna (Suppl. material 2) and the number of recorded species has expanded to 371 (own data). Although the fauna of the country is far from fully documented, we did not find new species, most probably because the Mecsek Mts. and its surroundings are amongst the better studied areas (Móra and Dévai 2004, Méhes et al. 2012, Szivák et al. 2013, Boóz and Móra 2020), but we provided new occurrence data for many rare species.

In addition to species, morphotypes without clear taxonomic state (see Notes on selected taxa) have also been found in each country, with the possibility that some of them might be new to science. Our results are in concordance with the conclusion that undescribed species are still being discovered, even in well-documented areas like the Western Palearctic Region (Whitmore et al. 2021) and, in the taxonomy of many groups remain unresolved even within Europe (Ekrem et al. 2010).

Although our study focused on small streams representing a limited range of habitat types, a remarkably high number of taxa have been detected. It might be due to the extensive sampling and accurate sorting process that increase the chance to find rare species (Boóz et al. 2024b). Additionally, the multi-level subsampling (Chimeno et al. 2023) for detailed species-level identification, which is frequently neglected in ecological, biodiversity or biomonitoring research (e.g. Raunio et al. (2011), Dorić et al. (2021)), increased remarkably the number of detected taxa.

Using DNA barcoding is an effective method for species identification; however, its success largely depends on the availability of appropriate reference databases (Ekrem et al. 2007, Mrozińska and Obolewski 2024). With the increasing effort in barcoding campaigns, more data are becoming available for Chironomidae. However, reference databases of the Central European Chironomidae are incomplete, as the number of public barcodes from our study area demonstrates: from Czechia, 21 COI-sequences are available, while the number of public barcodes is five from both Hungary and Croatia. In addition, we found some inconsistencies in molecular results (e.g. *Zavrelimyia* species, see Notes on selected taxa) and these did not fit with the results based on morphological investigation in some cases, highlighting the conspicuous gaps in the databases. It is widely recognised that combining traditional taxonomy with molecular tools can contribute to the better documentation of a region’s Chironomidae fauna (Montagna et al. 2016) and, accordingly, DNA barcoding has contributed to our taxa list in a few cases.

Finally, we can conclude that extensive studies in relatively small areas with a limited range of habitats (only streams in hilly regions) can remarkably contribute to the local and global knowledge on Chironomidae fauna, especially when taxonomically difficult and often problematic larvae are investigated. However, the number of species might increase and taxonomic problems might be solved more easily, if all life stages and molecular methods are included in these studies.

## Supplementary Material

XML Treatment for Chironomidae

CA65F133-4DAD-5ED6-8B80-E6F95C0160D9 10.3897/BDJ.12.e136241.suppl1 Supplementary material 1 Supplement 1 Data type description of the methods File: oo_1193742.docx https://binary.pensoft.net/file/1193742 Bernadett Boóz, Zsolt Kovács, Bea Bartalovics, Pál Boda, Marko Miliša, Bálint Pernecker, Petr Pařil, Tomasz Rewicz, Anna Boglárka Simon, Zoltán Csabai, Arnold Móra

20646B7F-610F-50D4-8252-C3AB3C182489 10.3897/BDJ.12.e136241.suppl2 Supplementary material 2 Supplement 2 Data type references Brief description Last comprehensive check-list of Chironomidae of Czechia, Croatia and Hungary and the papers with newly-recorded species published since then. File: oo_1123626.docx https://binary.pensoft.net/file/1123626 Bernadett Boóz, Zsolt Kovács, Bea Bartalovics, Pál Boda, Marko Miliša, Bálint Pernecker, Petr Pařil, Tomasz Rewicz, Anna Boglárka Simon, Zoltán Csabai, Arnold Móra
