# Supplementary material for: Chironomids (Diptera) from Central European stream networks: new findings and taxonomic issues
Source: Biodivers Data J. 2024 Dec 27;12:e136241. doi: 10.3897/BDJ.12.e136241 (PMC11699512; doi:10.3897/BDJ.12.e136241)
Supplement: Supplementary material 1 — Supplement 1 [file bdj-12-e136241-s001.docx]

**Supplement 1. Detailed descriptions of methods**

Sampling:

In Bükkösdi-víz catchment quantitative samplings were carried out through seasonal sampling campaigns from autumn 2018 to summer 2019. Multihabitat sampling protocol was applied within a 100-meter-long stream reach, with 12 collected subsamples (cca. 0.75 m^2^ sampled area) using kick & sweep method. In 2021, six bimonthly repeated sampling series were conducted in all three catchment areas. In these campaigns the mean wetted width of the stream/pools determined the sampled reach's length (20 × wetted width, 50–150 m) and the sampled area (0.5–1.5 m^2^ in flowing conditions, 0.1–0.5 m^2^ when only pools were present). Quantitative samples were taken using a Surber net. Samples were preserved in 96% ethanol, then sorting of collected specimens took place in the laboratory using stereomicroscopes. Additionally, at some sites in Bükkösdi-víz catchment, floating chironomid pupal exuviae were collected from the water surface using hand net and plastic tray in 2021.

Sample processing:

According to Bouchard and Ferrington (2011), 300 chironomid pupal exuviae is sufficient to capture approximately 85% of the total taxa richness. We applied this threshold for chironomid larvae too: when the samples contained less than 300 individuals, all of them were identified, while in the case of bigger samples about 300 individuals were subsampled. We aimed to achieve species-level identification but the identification of the larval specimens of Chironomidae is often only possible to genus-level due to the lack of appropriate keys and many species have unknown larval stage. Accordingly, species were not identified in the following genera: *Bryophaenocladius,* *Cryptochironomus, Cryptotendipes, Gymnometriocnemus, Procladius (Holotanypus), Pseudosmittia, Smittia, Tanytarsus* (except *T. triangularis* group), *Thienemanniella* and *Xenopelopia*. In some genera, larvae are not separable, but species can be identified as pupae or exuviae (*Conchapelopia, Krenopsectra, Micropsectra, Natarsia, Neozavrelia, Pseudorthocladius, Rheopelopia, Rheotanytarsus, Trissopelopia*). Further details can be found in the “Notes on selected taxa” section.

In the case of samples taken in Hungary during the 2018/2019 period, larvae were initially grouped into morphotypes based on macroscopic characters. Thereafter, a subset of individuals (typically 10-30 specimens for morphotypes with a higher number of individuals) underwent maceration process and were mounted on permanent slides to facilitate identification and to verify the number of species within a morphotype group (Chimeno et al. 2023). As for the 2021 collections, due to the large number of samples and individuals, identification of larvae mainly relied on macroscopic characters, resulting in genus, species group, or species level data, whenever it was possible using a stereomicroscope (magnification up to 35×). In some cases, when morphological characters were not easily observable and a higher optical resolution was required, a light microscope (magnification up to 400×) was used without extensive preparation (Orendt and Bendt 2021). Only the subset of some selected specimens, that were difficult to address this way, underwent a maceration process and were mounted on permanent slides for examination at higher magnification. The pupae were processed similarly, and only pupal exuviae were mounted on microscope slides in every instance. Selected specimens are deposited at the University of Pécs, Hungary.

Identification:

For the morphological identification of larvae, the following keys were used: Hirvenoja (1973), Cranston (1982), Kiknadze (1991), Schmid (1993), Janecek (1998), Sæther et al. (2000), Klink and Moller Pillot (2003), Ekrem (2004), Stur and Ekrem (2006), Vallenduuk and Moller Pillot (2007), Orendt and Spies (2012), Andersen et al. (2013), Rossaro and Lencioni (2015), Vallenduuk (2017), Cuppen and Tempelman (2018), Orendt and Bendt (2021), Cuppen and Tempelman (2022). For identification of pupae and exuviae keys by Michiels and Spies (2002), Langton and Visser (2003), Ekrem (2004) and Stur and Ekrem (2006) were used.

Molecular analysis:

In addition to morphological identification, a subset of larvae underwent molecular analysis using traditional DNA barcoding protocol. Genomic DNA was extracted from the abdomen muscle, while the head, thorax, and the posterior part of the specimens were preserved for later morphological analysis. DNA extraction was performed by using a Genomic Mini instant kit (A&A Biotechnology, Gdansk, Poland) at the University of Lodz, Łódź, Poland, following the manufacturer's protocol.

The initial amplification, quality control, and sequencing of the standard gene region used for animal DNA barcoding (COI—cytochrome c oxidase subunit I) followed the protocols in Srivatshan et al. (2021, 2024) using Oxford Nanopore Technology (ONT). COI was amplified using the primer pair LCO1490-JJ and HCO2198-JJ (Astrin and Stüben 2008), each tagged with 9 bp identifiers as specified in Srivatshan et al. (2024). Sequencing results were demultiplexed, and DNA barcodes were generated using ONT Barcoder 2.0 (Srivatshan et al. 2024). To improve amplification and sequencing success for samples that initially failed, we performed a second round of amplification using a primer cocktail consisting of LCO1490-JJ/HCO2198-JJ and LEPF1/LEPR1 (Hebert et al. 2004). Reaction conditions for this step adhered to those outlined in Hebert et al. (2004). PCR amplifications were conducted in 12 μl reactions comprising DreamTaq reaction buffer, 5 μmol of each primer, ultrapure water, and DNA template. Amplification success was assessed by checking 2 μL of each reaction product via 1% agarose gel electrophoresis. Bi-directional Sanger sequencing was outsourced to Macrogen Europe (Amsterdam, the Netherlands). The resulting sequences were edited, trimmed to remove primers, and aligned using the Geneious 11.1.5 software package (Kearse et al. 2012).

All sequences were uploaded to the Barcode of Life Data System (BOLD; Ratnasingham and Hebert 2007). Additionally, sequences were deposited in a separate DS-CHICE dataset, where all the relevant metadata are publicly available upon publication (<https://v4.boldsystems.org/index.php/MAS_Management_DataConsole?codes=DS-CHICE>). Sequences ≥300 base pairs (bp) were automatically assigned a Barcode Index Number. Sequences ≥500 bp, which did not find a match, served as founders of unique BINs. Voucher specimens are deposited at the University of Pécs, Hungary.

References:

Andersen T, Cranston PS, Epler JH (2013) The larvae of Chironomidae (Diptera) of the Holarctic Region – Keys and diagnoses*.* Insect Systematics and Evolution Supplements 66: 1-573.

Astrin JJ, Stüben PE (2008) Phylogeny in cryptic weevils: molecules, morphology and new genera of western Palaearctic Cryptorhynchinae (Coleoptera: Curculionidae). Invertebrate systematics 22: 503-522.

Bouchard RW, Ferrington LC (2011) The effects of subsampling and sampling frequency on the use of surface-floating pupal exuviae to measure Chironomidae (Diptera) communities in wadeable temperate streams. Environmental Monitoring and Assessment 181: 205‑223.<https://doi.org/10.1007/s10661-010-1824-6>

Chimeno C, Rulik B, Manfrin A, Kalinkat G, Hölker F, Baranov V (2023) Facing the infinity: tackling large samples of challenging Chironomidae (Diptera) with an integrative approach. PeerJ 11: e15336.<https://doi.org/10.7717/peerj.15336>

Cranston PS (1982) A key to the larvae of the British Orthocladiinae (Chironomidae). Freshwater Biological Association Scientific publication 45: 1‑152.

Cuppen H, Tempelman D (2018) Identification key for the 4th stage larvae of northwest European species of *Cricotopus* (Diptera: Chironomidae: Orthocladiinae). Lauterbornia 85: 69-90.

Cuppen H, Tempelman D (2022) Identification keys for the 4th stage larvae and pupal exuviae of north west European species of *Orthocladius* Van der Wulp, 1874 (Diptera: Chironomidae: Orthocladiinae). Lauterbornia 88: 83-124.

Ekrem T (2004) Immature stages of European Tanytarsus species I. The eminulus‐, gregarius‐, lugens‐ and mendax species groups (Diptera, Chironomidae). Deutsche entomologische Zeitschrift 51: 97‑146.<https://doi.org/10.1002/mmnd.20040510110>

Folmer O, Black M, Hoeh W, Lutz R, Vrijenhoek R (1994) DNA primers for amplification of mitochondrial cytochrome c oxidase subunit I from diverse metazoan invertebrates. Molecular Marine Biology and Biotechnology 3: 294‑299.

Janecek BFR (1998) Diptera: Chironomidae (Zuckmücken). Bestimmung von 4. Larvenstadien mitteleuropäischer Gattungen und österreichischer Arten. In: Fauna Aquatica Austriaca V. Kursmaterial, Universität für Bodenkultur, Abteilung Hydrobiologie, Wien.

Hebert PD, Penton EH, Burns JM, Janzen DH, Hallwachs W (2004) Ten species in one: DNA barcoding reveals cryptic species in the neotropical skipper butterfly Astraptes fulgerator. Proceedings of the National Academy of Sciences 101: 14812-14817.

Hirvenoja M (1973) Revision der Gattung Cricotopus van der Wulp und ihrer Verwandten (Diptera, Chironomidae). Annales Zoologici Fennici 10: 1‑363.

Hou Z, Fu J, Li S (2007) A molecular phylogeny of the genus Gammarus (Crustacea: Amphipoda) based on mitochondrial and nuclear gene sequences. Molecular Phylogenetics and Evolution 45: 596-611.<https://doi.org/10.1016/j.ympev.2007.06.006>

Kearse M, Moir R, Wilson A, Stones-Havas S, Cheung M, Sturrock S, Buxton S, Cooper A, Markowitz S, Duran C, Thierer T, Ashton B, Meintjes P, Drummond A (2012) Geneious Basic: an integrated and extendable desktop software platform for the organization and analysis of sequence data. Bioinformatics 28: 1647‑1649.<https://doi.org/10.1093/bioinformatics/bts199>

Kiknadze II (1991) Karyotypes and larval morphology in tribe Chironomini. An Atlas. Novosibirsk: Nauka. 113 p.

Klink AG, Moller Pillot HKM (2003) Chironomidae larvae. Key to the higher taxa and species of the lowlands of Northwestern Europe. In: World Biodiversity Database CD-ROM Series. – Multimedia Interactive Software 1.0., Expert Center for Taxonomic Identification, University of Amsterdam, Amsterdam.

Langton PH, Visser H (2003) Chironomidae exuviae. Key to pupal exuviae of the West Palaearctic Region. In: World Biodiversity Database CD-ROM Series. – Multimedia Interactive Software 1.0., Expert Center for Taxonomic Identification, University of Amsterdam, Amsterdam

Michiels S, Spies M (2002) Description of *Conchapelopia hittmairorum*, spec. nov., and redefinition of similar western Palaearctic species (Insecta, Diptera, Chironomidae, Tanypodinae). Spixiana 25: 251-272.

Orendt C, Spies M (2012) Chironomus Meigen (Diptera: Chironomidae). Key to the larvae of importance to biological water analysis in Germany and adjacent areas. Orendt Hydrobiologie, Liepzig, 24 pp.

Orendt C, Bendt T (2021) Orthocladiinae sensu lato (Orthocladiinae, Prodiamesinae, Diamesinae, Podonominae, Buchonomyiinae, Telmatogetoninae) (Diptera: Chironomidae). Keys to Central European larvae with respect to macroscopic characters. Deutsche Gesellschaft für Limnologie e.V., 144 pp.

Ratnasingham S, Hebert PDN (2007) BOLD: The Barcode of Life Data System (http://www.barcodinglife.org). Molecular Ecology Notes 7: 355-364.<https://doi.org/10.1111/j.1471-8286.2007.01678.x>

Rossaro B, Lencioni V (2015) A key to larvae of Diamesa Meigen, 1835 (Diptera, Chironomidae), well known as adult males and pupae from Alps (Europe). Journal of Entomological and Acarological Research 47: 5516.<https://doi.org/10.4081/jear.2015.5516>

Sæther OA, Ashe P, Murray DA (2000) A.6. Family Chironomidae. In: Papp L, Darvas B (Eds) Contribution to a manual of Palaearctic Diptera. Appendix. Science Herald, Budapest, 113-334 pp.

Schmid PE (1993) A key to the larval Chironomidae and their instars from Austrian Danube Region streams and rivers, with particular reference to a numerical taxonomic approach. Part I. Diamesinae, Prodiamesinae and Orthocladiinae. 3/93. Wasser und Abwasser Supplement, 514 pp.

Srivathsan A. Lee L, Katoh K, Hartop E, Kutty SN, Wong J, Darren Y, Meier R (2021) ONTbarcoder and MinION barcodes aid biodiversity discovery and identification by everyone, for everyone. BMC biology 19: 1-21.

Srivathsan A, Feng V, Suárez D, Emerson B, Meier, R (2024) ONTbarcoder 2.0: rapid species discovery and identification with real‐time barcoding facilitated by Oxford Nanopore R10. 4. Cladistics 40: 192-203.

Stur E, Ekrem T (2006) A revision of West Palaearctic species of the Micropsectra atrofasciata species group (Diptera: Chironomidae). Zoological Journal of the Linnean Society 146: 165‑225.<https://doi.org/10.1111/j.1096-3642.2006.00198.x>

Vallenduuk HJ, Moller Pillot HKM (2007) Chironomidae larvae. General ecology and Tanypodinae. KNNV Publishing, Zeist, 144 pp.

Vallenduuk HJ (2017) Chironomini larvae of western European lowlands (Diptera: Chironomidae). Keys with notes to the species. With a redescription of Glyptotendipes (Caulochironomus) nagorskayae and a first description of Glyptotendipes (Caulochironomus) kaluginae new species. Lauterbornia 82: 1-216.
