# Supplementary material for: Chironomids (Diptera) from Central European stream networks: new findings and taxonomic issues
Source: Biodivers Data J. 2024 Dec 27;12:e136241. doi: 10.3897/BDJ.12.e136241 (PMC11699512; doi:10.3897/BDJ.12.e136241)
Supplement: Supplementary material 2 — Supplement 2 [file bdj-12-e136241-s002.docx]

**Supplement 2. Last comprehensive check-list of Chironomidae of Czechia, Croatia and Hungary, and the papers with newly recorded species published since then**

**Croatia**

Čerba D, Koh M, Ergović V, Mihaljević Z, Milošević D, Hamerlík L (2020) Chironomidae (Diptera) of Croatia with notes on the diversity and distribution in various habitat types. Zootaxa 4780: 259-274.<https://doi.org/10.11646/zootaxa.4780.2.2>

Čerba D, Vlaičević B, Davidović RA, Koh M, Ergović V, Turković Čakalić I (2023) Chironomidae in shallow water bodies of a protected lowland freshwater floodplain ecosystem. Science Progress 106(2),<https://doi.org/10.1177/00368504231172653>

Dorić V, Pozojević I, Baranov V, Mihaljević Z, Ivković M (2024) Long-term chironomid emergence at a karst tufa barrier in Plitvice Lakes National Park, Croatia. Insects 15: 51.<https://doi.org/10.3390/insects15010051>

Ergović V, Koh M, Čerba D, Mihaljević Z, Hamerlík L (2023) Evidence of new chironomid taxa (Diptera, Chironomidae) for Croatia from a mountain stream in the Pannonian Plain. Ecologica Montenegrina 70: 128-136.<https://doi.org/10.37828/em.2023.70.14>

**Czechia**

Bitušík P, Brabec K (2009) Chironomidae Newman, 1834. Checklist of Diptera of the Czech Republic and Slovakia. Electronic version, 2.<http://www.edvis.sk/diptera2009/families/chironomidae.htm>(accessed 28.08.2024)

Ashe P, Moubayed-Breil J, Vondrák D (2014) First records of *Buchonomyia thienemanni* Fittkau (Diptera: Chironomidae) from the Czech Republic. CHIRONOMUS Journal of Chironomidae Research 27: 51-53.<https://doi.org/10.5324/cjcr.v0i27.1711>

Bitušík P, Trnková K (2016) Some new records of chironomids (Diptera: Chironomidae) from the Czech Republic. Acta Musei Silesiae Scientiae Natururales 65: 143-147.<https://doi.org/10.1515/cszma-2016-0018>

Orendt C, Wolfram G, Adámek Z, Jurajda P, Schmitt-Jansen M (2012) The response of macroinvertebrate community taxa and functional groups to pollution along a heavily impacted river in Central Europe (Bílina River, Czech Republic). Biologia 67: 180-199.<https://doi.org/10.2478/s11756-011-0158-3>

Skála I (2011) Faunistic records from the Czech Republic – 318. Diptera: Chironomidae. Klapalekiana 47: 265-269.

Skála I (2012) Faunistic records from the Czech Republic – 331. Diptera: Chironomidae. Klapalekiana 48: 151-156.

Skála I (2013) Faunistic records from the Czech Republic – 347. Diptera: Chironomidae. Klapalekiana 49: 107-108.

Skála I (2014) Faunistic records from the Czech Republic – 372. Diptera: Chironomidae. Klapalekiana 50: 257-258.

Špaček J (2014) Faunisticky zajímavé nálezy vodních bezobratlých Krkonos [Faunistically important records of water invertebrates in the Giant Mts]. Opera Corcontica 51: 199-204.

Syrovátka V, Langton PH (2015) First records of *Lasiodiamesa gracilis* (Kieffer, 1924), *Parochlus kiefferi* (Garrett, 1925) and several other Chironomidae from the Czech Republic and Slovakia. CHIRONOMUS Journal of Chironomidae Research 28: 45-56.<https://doi.org/10.5324/cjcr.v0i28.1953>

**Hungary**

Móra A, Dévai G (2004) Magyarország árvaszúnyog-faunájának (Diptera: Chironomidae) jegyzéke az előfordulási adatok és sajátosságok feltüntetésével. Acta Biologica Debrecina Supplementum Oecologica Hungarica 12: 39-207.

Andrikovics S, Kiss O, Nagy B (2005) Hosszú és rövid periódusú változásokról a Szalajka-patak gerinctelen makrofauna közösségeiben (Bükk hegység, Magyarország). Acta Biologica Debrecina Supplementum Oecologica Hungarica 13: 9-19.

Árva D, Móra A, Tóth M, Nosek J (2011) A metafiton árvaszúnyoglárva-együttesei a Duna árterein (Béda−Karapancsa, Gemenc, Szigetköz). Acta Biologica Debrecina Supplementum Oecologica Hungarica 26: 9-20.

Boda P, Móra A, Csabai Z (2019) Aquatic macroinvertebrates from soda pans and adjacent wetland habitats of the Hungarian Puszta region with first records of four species from Hungary. Spixiana 42: 263-282.

Boda P, Móra A, Csabai Z (2023) AMI-KMNP dataset: Occurrence records of aquatic macroinvertebrate species from a 10-year-long biodiversity survey in SE Hungary. Journal of Limnology 82: 2118.<https://doi.org/10.4081/jlimnol.2023.2118>

Boóz B, Móra A (2020) Data on the Chironomidae (Diptera) fauna of streams originating from the Mecsek Mountains, SW Hungary. Natura Somogyiensis 35: 87-98.<https://doi.org/10.24394/NatSom.2020.35.87>

Csabai Z, Boda P, Boda R, Bódis E, Danyik T, Deák C, Farkas A, Kálmán Z, Lőkkös A, Málnás K, Mauchart P, Móra A (2015) Aquatic macroinvertebrate fauna of the Kis-Sárrét Nature Protection Area with first records of five species from Hungary. Acta Biologica Debrecina Supplementum Oecologica Hungarica 33: 9-70.

Csányi B, Graf W, Hartmann A, Huber T, Moog O, Stubauer I (2010) Rába vizsgálat 2009. Az ökológiai állapot vizsgálata a Rába hossz-szelvénye mentén. Makrozoobenton biológiai minőségi elem (BQE). Kutatási jelentés, Universität für Bodenkultur Wien – VITUKI Kft., Bécs, 89 pp. + Appendix

Csépes E, Tóth M, Móra A (2012) The chironomid fauna of the reservoir Kiskörei-tározó (Diptera: Chironomidae). Acta Biologica Debrecina Supplementum Oecologica Hungarica 27: 15-26.

Graf W, Csányi B, Leitner P, Paunović M, Janeček B, Šporka F, Chiriac G, Stubauer I, Ofenböck T (2008) Macroinvertebrates. Final report, full version. In: Joint Danube Survey 2. Final Scientific Report. International Commission for the Protection of the Danube River, Vienna, 87 pp. + Appendix on CD-ROM

Horváth Z, Móra A, Ambrus A, Szövényi G, Andrikovics S (2009) Makrogerinctelen-együttesek tér- és időbeli változásai a hansági Nyirkai-Hany élőhely-rekonstrukciós területen. Acta Biologica Debrecina Supplementum Oecologica Hungarica 20: 115-126.

Méhes N, Szivák I, Csabai Z, Móra A (2012) Contribution to the Chironomidae (Diptera) fauna of the Mecsek Mountains. Acta Biologica Debrecina Supplementum Oecologica Hungarica 28: 121-128.

Móra A (2014) Contribution to the Chironomidae (Diptera) fauna of Lake Balaton and its catchment area, with first records of nine species from Hungary. Acta Biologica Debrecina Supplementum Oecologica Hungarica 32: 107-127.

Móra A, Barnucz E, Boda P, Csabai Z, Cser B, Deák C, Papp L (2007) A Balaton környéki kisvízfolyások makroszkópikus gerinctelen faunája. Acta Biologica Debrecina Supplementum Oecologica Hungarica 16: 105-167.

Móra A, Bíró K (2007) Árvaszúnyogok (Diptera: Chironomidae) Magyarországról 4. Lárvák a Balatonba ömlő patakokból. Acta Biologica Debrecina Supplementum Oecologica Hungarica 16: 169-174.

Móra A, Boda P, Csabai Z, Cser B, Deák C, Hornyák A, Jakab T, Kálmán Z, Kecső K, Kovács TZ, Papp L, Polyák L, Soós N (2008) A Zala és befolyói makroszkopikus gerinctelen faunája. Acta Biologica Debrecina Supplementum Oecologica Hungarica 18: 123-180.

Móra A, Csabai Z (2019) Chapter 16. Aquatic macroinvertebrates of the Drava River and its floodplain. In: Lóczy D (ed.): The Drava River. Springer Geography, Springer, Cham, pp. 247-279.

Móra A, Deák C, Lőkkös A, Papp L, Soós N, Csabai Z (2010a) A Porva melletti Hódos-ér vízi makrogerinctelen faunájáról a 2008. évi Biodiverzitás Nap eredményei alapján. Folia Musei Historico-naturalis Bakonyiensis 27: 75-82.

Móra A, Farkas A (2012) The Chironomidae (Diptera) fauna of the Szentendrei-Duna, Hungary. Acta Biologica Debrecina Supplementum Oecologica Hungarica 28: 129-140.

Móra A, Kálmán Z, Soós N, Tóth A, Deák C, Ambrus A, Csabai Z (2010b) Data to the aquatic invertebrate fauna of Kis-Duna (Kismaros) with first Hungarian records of three chironomid species. Acta Biologica Debrecina Supplementum Oecologica Hungarica 21: 127-138.

Móra A, Polyák L, Farkas A (2013) Contribution to the Chironomidae (Diptera) fauna of the Sajó/Slaná River, Hungary and Slovakia. Acta Biologica Debrecina Supplementum Oecologica Hungarica 31: 69-81.

Móra A, Tóth M, Debreceni Á, Csépes E. (2006) Adatok a Felső-Tisza árvaszúnyog-faunájához (Diptera: Chironomidae). Folia Historico-naturalia Musei Matraensis 30: 253-261.

Nagy B, Kiss O, Andrikovics S (2007) A medertisztítás hatásairól a Szalajka-patakban. Természetvédelmi Közlemények 13: 289-298.

Oertel N, Nosek J, Andrikovics S (2005) A magyar Duna-szakasz litorális zónájának makroszkopikus gerinctelen faunája (1998-2000). Acta Biologica Debrecina Supplementum Oecologica Hungarica 13: 159-185.

Szító A, Bancsi I, Kovács P, Végvári P, Éhn J, Szalma E (2005) A duzzasztási szintek várható hatása a Hármas-Körösben az üledéklakó gerinctelen életközösségekre. Hidrológiai Közlöny 85/6: 134-137.
